# Umbrella review of systematic reviews to inform the development and translation of community‐based childhood obesity prevention interventions

**DOI:** 10.1111/obr.13864

**Published:** 2024-11-23

**Authors:** Jane Jacobs, Luke Wolfenden, Kristy A. Bolton, Vicki Brown, Marufa Sultana, Kathryn Backholer, Steven Allender, Rachel Novotny, Anna Peeters, Melanie Nichols

**Affiliations:** ^1^ Institute for Health Transformation, Global Centre for Preventive Health and Nutrition, School of Health and Social Development, Faculty of Health Deakin University Geelong Australia; ^2^ School of Medicine and Public Health, Faculty of Health and Medicine University of Newcastle Callaghan NSW Australia; ^3^ Institute for Physical Activity and Nutrition, School of Exercise and Nutrition Sciences, Faculty of Health Deakin University Geelong Australia; ^4^ Institute for Health Transformation, Deakin Health Economics, School of Health and Social Development, Faculty of Health Deakin University Geelong Australia; ^5^ College of Tropical Agriculture and Human Resources, Department of Human Nutrition, Food and Animal Sciences University of Hawaii at Manoa Honolulu Hawaii USA

**Keywords:** childhood obesity, community, interventions, umbrella review

## Abstract

Community‐based interventions (CBIs) can be effective and feasible for the prevention of childhood obesity. The aim of this umbrella review is to determine if systematic reviews report sufficient information to guide replication or adaptation of CBIs to a variety of contexts and aid in further development of childhood obesity prevention CBIs. Six databases were searched for systematic reviews including obesity prevention CBIs involving 0–18 year olds and reporting weight‐related outcomes. Two researchers screened results. Evidence‐to‐decision frameworks guided which details may be required for decision‐makers to design and carry‐out a CBI, including information on intervention characteristics, outcome reporting and translation factors. From 3935 search results, 40 studies were included. The most frequently reported relevant pieces of information were behaviors targeted (100% of systematic reviews), intervention duration (90%) and settings involved (97.5%). Less frequently reported factors included specific actions implemented (48%), intervention intensity (30%) and organizations, or contributors involved (40%). There was a low level of reporting of equity considerations (27.5%), adverse events (20%), and costs/cost‐effectiveness (17.5%). Multilevel interventions for child obesity prevention have demonstrated effectiveness, yet additional documentation of successful intervention processes is needed.

## 1 BACKGROUND

Overweight and obesity remain among the most prevalent and intractable child health problems in high‐income countries, and rapidly rising rates in low‐ and middle‐income countries are of increasing concern.^1^ The lifelong consequences of excess body fat during childhood represent a significant health, quality of life, and financial cost to individuals and societies.^2^, ^3^

Addressing childhood overweight and obesity requires comprehensive responses at multiple levels of governance and must address the many systems influencing population weight gain.^4^ Among the most promising responses, which have been investigated in many countries over the last two decades, are community‐based primary prevention interventions.^4^, ^5^ These interventions are typically multistrategy, multisetting programs of activity, engaging a wide range of community contributors, and are usually either community‐led, or flexible enough to be adapted to the context, resources and priorities of the local communities in which they are implemented.^4^, ^5^, ^6^ A number of these community‐based interventions (CBIs) have demonstrated beneficial impacts on child body mass index (BMI),^7^, ^8^, ^9^ weight‐related behaviors (dietary intake, physical activity, sedentary behaviors), and quality of life.^10^ Available economic evidence also suggests that they can be cost‐effective.^11^

Despite their promise, systematic reviews of CBIs report considerable heterogeneity in their effects. Published systematic reviews have summarized the weight‐related or behavioral outcomes of childhood obesity prevention interventions^12^, ^13^; however, few focus on what characteristics of CBIs have been associated with effectiveness, in what context, and for whom. This is particularly challenging in the field of community‐based obesity prevention, with a wide range of intervention characteristics and strategies, and varying contexts and populations in which the interventions have been trialed or implemented. Research to better understand the characteristics of CBIs, the contexts and population groups in which they may be most effective, is needed to improve their impact in practice and help inform prudent public health investment in such initiatives.

Systematic reviews, rather than only presenting the findings of individual studies, are recommended as the basis for evidence informed public health policy and practice. If interventions in communities, or reviews of these studies, are to improve population health, knowledge translation frameworks suggest they should be produced and reported in ways that align with the needs of these decision‐makers.^14^ There is increasing evidence that the published literature does not provide adequate detail on practice‐relevant^15^, ^16^ and policy‐relevant^15^, ^17^ aspects of obesity prevention interventions. A review of systematic reviews in 2010 by Wolfenden et al.^16^ found less than 50% of included publications reported intervention effects by settings or intervention modalities and very few reported costs or adverse events associated with interventions. Similarly, a review of policy‐related systematic reviews by Kite et al.^17^ found that less than a third of the included publications included discussions related to policy implications of their findings. This limits the ability of decision‐makers to appraise the potential appropriateness and impact of CBIs if implemented in their context. Such information is also needed to sufficiently inform advancement of the field of research, to optimize future interventions, and to understand and avoid repetition of ineffective approaches.

The question of what makes a review ‘useful’ or relevant to decision‐makers is not a simple one. The key types of information, and their relative importance, will be different for a range of different organizations or communities, and may vary over time and across contexts. The Grading of Recommendations Assessment, Development and Evaluation (GRADE) evidence to decision framework was developed to provide guidance on the use of evidence in a structured manner in order to guide decision‐making across a variety of contexts.^18^, ^19^ Further building on this framework, the World Health Organization Integrate Evidence (WHO‐INTEGRATE) framework^14^ was developed to ensure alignment with the WHO values and norms and is particularly valuable for complex population and system‐level interventions. Another tool that can assist in the evaluation of complex interventions is the Intervention Complexity Assessment Tool for Systematic Review (iCAT_SR), which facilitates the assessment of the complexity of interventions evaluated by systematic reviews.^20^ These can be helpful in determining what information should be reported in trials and synthesized in systematic reviews in order for them to be useful for decision‐makers.

In this umbrella review, we sought to investigate whether published systematic reviews report sufficient information to guide replication or adaptation of CBIs to a variety of contexts and aid in further development of childhood obesity prevention CBIs.

Specifically, we aimed to evaluate the extent to which published systematic reviews report and analyze:

1. Intervention characteristics that describe what the intervention entailed, who designed and delivered it, in what setting, and for how long;
2. Outcomes that demonstrate the effectiveness, sustainability, potential harms, and equity considerations of CBIs and;
3. Translation factors that would be useful for determining external validity and replicability.

## 2 METHODS

An umbrella review was chosen to ensure a rigorous and comprehensive approach to our search strategy and reporting methods in order to address our aims.^21^ This umbrella review was registered with PROSPERO (CRD42022335472) on August 13, 2022. Guidelines for the reporting of umbrella reviews are currently being developed.^22^ Therefore, the search, study selection, analysis, and reporting of results were undertaken using the relevant aspects of the Preferred Reporting Items for Overview of Systematic Reviews (PRIO‐harms) checklist.^23^

### 2.1 Search strategy

We searched six databases (Medline Complete, CINAHL, EMBASE, Cochrane Database of Systematic Reviews, Database of Abstracts of Reviews and Effects, PROSPERO) using five key concepts of “systematic review or meta‐analysis,” “intervention,” “prevention,” “obesity,” and “children or adolescents.” Key terms and MESH headings were used and adapted for each database. The search was limited to publications in English and post 1994. This date was based on previous work by Wolfenden et al.,^16^ who in 2010 conducted a review of reviews across all child obesity interventions and found no relevant systematic reviews prior to 1994. The full search strategy is provided in Table S1. The initial search was completed on July 27, 2022, and an updated search completed on June 15, 2023.

### 2.2 Study selection

Papers were included if they were a systematic review (with or without meta‐analysis) that included intervention studies focused on childhood obesity prevention. Based on work by Martinic et al.,^24^ to be considered a systematic review, studies needed to (i) have a clear research question, (ii) identify sources searched and a reproducible search strategy, (iii) state inclusion/exclusion criteria, (iv) identify method of screening at each stage, (v) critically appraise the included studies, and (vi) include information on data analysis and synthesis.

Other inclusion criteria for reviews were (i) included obesity prevention CBIs and (ii) presented weight‐related results (e.g., BMI, waist circumference) for children or adolescents (included ages 0–18 years). We define CBIs as multicomponent primary prevention interventions implemented either in schools or in two or more other community settings (e.g. health services, community/recreation centers, local governments).^6^ School‐only interventions were included as literature on community engagement defines a school as a ‘community’.^25^ If a systematic review did not focus exclusively on studies meeting this definition for CBIs, it was still eligible for inclusion if results for child‐ or adolescent‐focused CBIs were collated and presented as a defined stratum or group. Where a systematic review was an update of a previously published review and the updated version wholly overlapped with the older paper, the older paper was excluded to minimize duplication.

Search results identified from each database were loaded into Covidence^26^ and duplicates removed. Two researchers (JJ and KBo) screened all papers by title and abstract, according to inclusion and exclusion criteria. Full text versions of potentially relevant studies were screened by the same two researchers. Any discrepancies were resolved through discussion between screeners and one additional researcher (MN) if required.

### 2.3 Data extraction

Data were extracted into a standardized data extraction spreadsheet. One researcher (JJ) completed data extraction for all included studies, with 20% also extracted by a second researcher (MN) to check for accuracy. Data were extracted under the two broad categories of “general” and “practice‐relevant” study information. General information included the following: author, year, total number of studies included, number of relevant studies included (prevention/community‐based/children), year limits, type of synthesis, inclusion criteria (population, intervention, comparator, primary outcome/s, intermediate outcome/s, study designs), and main conclusions.

Practice‐relevant information was broken into three domains: intervention characteristics; outcome reporting; and translation, replicability, and validity. Domains were identified based on two evidence to decision frameworks (GRADE^18^ and WHO‐INTEGRATE^14^) and a recent tool developed to assess intervention complexity of systematic reviews^20^ (Table 1).

**TABLE 1:** Origin of domains and characteristics for reporting.

Practice‐relevant data extracted from included reviews	WHO‐INTEGRATE^14^	Lewin (iCAT_SR)^20^	GRADE^18^
*Intervention characteristics – reported and/or analyzed*			
Behaviors targeted	Y	Y	
Specific strategies employed	Y	Y	
Duration	Y	Y	
Intensity	Y	Y	
Number or range of settings involved in interventions		Y	
Organizations or contributors involved in the intervention		Y	
*Outcome reporting*			
Intervention effectiveness summarized across studies			Y
Harms, or adverse effects	Y		Y
Sustainability of the intervention			Y
Equity impacts and considerations	Y		Y
*Translation, replicability and validity*			
Characteristics of the ‘community’ (e.g., SEP and rurality)	Y		
Characteristics of the participants	Y		
Quality assessment of included studies	Y		
Required resources or intervention costs reported	Y		Y

Abbreviations: GRADE, Grading of Recommendations Assessment, Development, and Evaluation; iCAT‐SR, Intervention Complexity Assessment Tool for Systematic Review; WHO‐INTEGRATE, World Health Organization Integrate Evidence.

Intervention characteristics included whether there was reporting and/or analysis in the review of the following: targeted behaviors; specific strategies or elements employed; duration of intervention; intensity of intervention; settings involved; and contributors or organizations involved. The outcome reporting domain included whether intervention effectiveness was summarized across studies (narratively or quantitatively); reporting of equity impacts or considerations (e.g., differential reporting across socio‐economic groups, cultural groups, rural–urban); reporting of harms or adverse effects; and sustainability of the intervention (reporting of outcomes at end of intervention, and at a later timepoint after intervention completed to assess if any effects were sustained in the absence of active intervention). The translation, replicability, and validity domain extracted information on whether the review included the following: characteristics of the “community” (e.g., location, area‐level socio‐economic position [SEP], and rurality); characteristics of the sample (e.g., sex, individual level SEP, and ethnicity); and resources used or intervention costs and/or cost‐effectiveness. Further details on each category are provided in Table S2.

The AMSTAR‐2 (A MeaSurement Tool to Assess systematic Reviews) quality checklist for systematic reviews^27^ was used to assess the quality of included systematic reviews. The checklist was completed by one researcher (JJ), with a 10% sample duplicated by a second researcher (MS), with any discrepancies resolved through discussion. The AMSTAR‐2 checklist includes seven “critical” and nine “noncritical” items and was developed to critically appraise systematic reviews of both randomized and non‐randomized studies. A systematic review is considered high quality if it includes no more than one noncritical weakness; moderate quality if it includes more than one noncritical weakness (but no critical weaknesses); low quality if it includes one critical weakness with or without noncritical weaknesses; and critically low if it includes more than one critical weakness, with or without noncritical weaknesses.^27^

### 2.4 Synthesis

A narrative synthesis was undertaken, with a focus on the reporting of key information within the three practice‐relevant domains outlined above.

## 3 RESULTS

**FIGURE 1:**
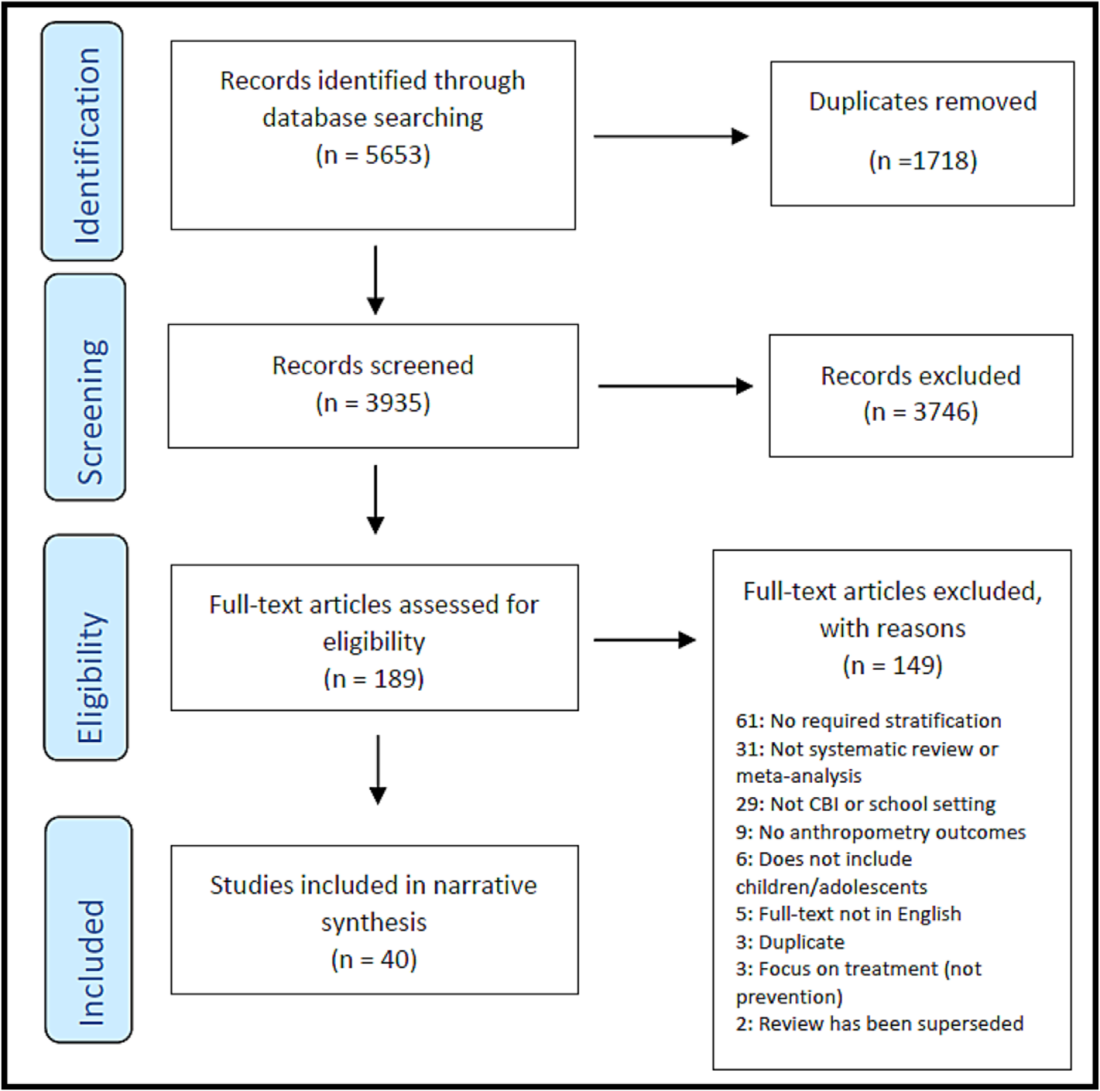
PRISMA flow‐chart.

The initial search identified 5653 publications, which reduced to 3935 after the removal of duplicates (Figure 1). Following title and abstract screening 198 potentially relevant reviews remained, with 40 reviews meeting the inclusion criteria after full‐text screening. The most common reasons for exclusion at the full text stage were that the reviews did not report a relevant stratification of child‐focused CBI outcomes (*n* = 61), did not meet the criteria for being a systematic review or meta‐analysis (*n* = 31), contained no primary studies that met the criteria for CBIs (*n* = 29) and did not present weight‐related outcomes (*n* = 9).

Included systematic reviews were published between 2001 and 2023. The number of included primary studies ranged from eight to 195, with those considered relevant to this umbrella review ranging from three to 88. Eleven reviews specified body mass index (BMI) or BMI *z* score as the required outcome measure, whereas the remaining studies required any measure of adiposity (e.g., BMI, fat mass index, and waist circumference) to assess effectiveness of the interventions. Behavioral outcomes including physical activity levels (such as meeting national guidelines, or time in moderate to vigorous physical activity) and dietary outcomes (such as fruit and vegetable consumption, or discretionary food intake) were also reported. The most common research designs used in the primary studies included in the systematic reviews were randomized and nonrandomized controlled trials and pre‐posttest designs. “General information” of included systematic reviews is included in Table 2.

**TABLE 2:** General information extracted from included systematic reviews (*n* = 40).

Author, year	Total studies (relevant studies)^a^	Year limits	Type of synthesis	Population	Intervention	Comparator	Primary outcome/s *(Other outcome/s)*	Study designs	Main conclusions
Angawi, 2021^28^	32 (21)	2010–2020	Narrative synthesis	2–19 year olds	Home‐based, school‐based, community‐based intervention; aim of avoiding or controlling weight gain; focus on PA or diet or both	Not defined	Any anthropometric outcomes *(dietary intake, PA, sedentary behaviors)*	RCT	Schools should be considered as primary setting for interventions to prevent childhood obesity, and incorporating diet and PA.
Bleich, 2013^12^	9 (9)	Inception to 2012	Narrative synthesis	2–18 year olds; high income countries	Obesity prevention; >1 year follow‐up; community settings	Usual care, another different intervention or no intervention	Any anthropometric outcomes	RCT; quasi‐experimental; natural experiments	Moderate strength of evidence indicates combined diet and PA intervention conducted in the community, with a school component can prevent obesity/overweight.
Bleich, 2018^29^	56(40)	2013–2017	Narrative synthesis	2–19 years	School, preschool, community or home settings; targeting PA and or diet; follow‐up ≥12 months (≥6 months for school setting)	Required control group, but not defined	Any anthropometric outcomes	RCT; quasi‐experimental; natural experiments with control group	School‐based interventions combining diet and PA hold promise for childhood obesity prevention.
Bramante, 2019^30^	33 (8)	2000–2017	Narrative synthesis	‘pediatric’ population (ages not specified)	Natural experiments; program or policy aimed at combating pediatric obesity	Required control group, but not defined	BMI, BMI *z* score, BMI percentile, BMI classification *(dietary intake, PA)*	Natural experiments	Natural experiments evaluating school‐based policies focusing on food and PA environments (compared with only one) showed BMI improvement.
Brown, 2009^31^	38 (20)		Narrative synthesis	5–18 year olds	Lifestyle intervention set in schools; ≥12 weeks duration	Usual care or other active intervention	Any anthropometric outcomes	RCT; controlled clinical trial	Findings inconsistent, but indicated that combined diet and PA. School‐based interventions could prevent children becoming overweight in the long term.
Brown, 2015^32^	29 (5)	2006–2014	Meta‐analysis and narrative synthesis	South Asian ethnicity (subanalysis of 0–18 year olds)	Any lifestyle intervention; any length of follow‐up, aim of managing obesity/obesity related diseases	Did not require control group	Any anthropometric outcomes	RCT; controlled clinical trial; before and after studies	No effects of interventions on BMI for South Asian children, from a small number of controlled trials.
Brown, 2016^33^	17 (17)	2007–2016	Narrative synthesis	Elementary school (ages not defined)	School based; includes PA and healthy eating components;	Control group, no intervention	BMI, BMI *z* score *(dietary intake, PA, sedentary behaviors)*	RCT; non‐RCT with no‐intervention controls	School‐based interventions may be an effective means for child obesity prevention.
Brown, 2019^34^	153 (36)	1990–2015	Meta‐analysis and narrative synthesis	Mean age of less than 18 at baseline	Diet and or PA interventions; preventing overweight or obesity in children; duration ≥12 weeks	No interventions, usual care or wait list comparisons	BMI, BMI *z* score *(adverse events)*	RCT	Interventions including PA alone can reduce the obesity risk in 6–12 and 13–18 year olds. Some evidence that combined diet and PA interventions may be effective. Interventions to prevent childhood obesity do not appear to result in adverse effects or health inequalities.
Buchanan, 2023^35^	24 (16)	1990–2019	Meta‐analysis and narrative synthesis	Kindergarten to high school	Intervention programs or policies aimed at school meals and/or fruit/veg snack consumption; included PA component.	Not specified	Any anthropometric outcomes *(dietary intake, PA)*	RCT; quasi‐experimental (with comparison); time series, before/after	Modest reduction in the prevalence of overweight and obesity for elementary students found for school meal or fruit/vegetable snack interventions combined with PA.
Campbell, 2001^36^	7 (3)	1985–1999	Narrative synthesis	0–18 year olds	Intervention study aim of preventing childhood obesity; Duration ≥3 months	Required control group (not defined)	Any anthropometric outcomes	RCT; non‐RCT with a concurrent control group	No generalizable conclusions due to limited quality data on the effectiveness of obesity prevention programs.
Cerrato‐Carretero, 2021^37^	11 (11)	2009–2019	Meta – analysis and narrative synthesis	6–12 year olds	School‐based; PA plus diet/dietary education; ≥1 academic year	No treatment, routine care; active intervention	BMI	RCT	Long‐term school‐based interventions on PA and dietary habits received by children aged 6–12 years had no effect on BMI.
Chavez, 2020^38^	16 (10)	2000–2017	Meta‐analysis and narrative synthesis	6–18 year olds; conducted in Latin American countries	Primary prevention of obesity; at least one school‐based component	Not defined	Any anthropometric outcomes *(dietary intake, PA)*	Controlled or before and after design	Most effective interventions had moderate quality and included multicomponent school‐based programs promoting health education and parental involvement focused on healthy eating and PA.
Connelly, 2007^39^	28 (15)	Inception to 2006	Narrative synthesis	0–18 years; nonoverweight children with or without overweight/obese children	Interventions to prevent overweight/obesity; with ≥12 week follow‐up	Not defined	Any anthropometric outcomes	RCT; non‐RCT	“Compulsory” provision of aerobic PA played decisive role in effectiveness
Dabravolskaj, 2020^40^	66 (47)	2012–2020	Meta‐analysis and narrative synthesis	4–18 year olds; conducted in countries with human development index ≥0.8	School based interventions; aim of preventing obesity and associated risk factors; ≥6 months duration	Not defined	BMI, BMI *z* score, percent overweight/obese *(fruit/veg intake, PA)*	Comparative studies	Decision‐makers should consider Comprehensive School Health (Canadian program) multicomponent interventions, modifications to PE curricula and school nutrition policies to prevent childhood obesity.
De Bourdeaudhuij, 2011^41^	11 (11)	1990–2007	Narrative synthesis	6–18 year olds; conducted in Europe	School‐based interventions; aim of primary prevention of obesity/obesity‐related disease; combination healthy diet and PA	Not defined	Any anthropometric outcome *(dietary intake, PA)*	Study design not limited,	Combining educational and environmental components that focus on both sides of the energy balance give better and more relevant effects.
Gao, 2008^42^	20 (6)	Inception to 2006	Narrative synthesis	Conducted in China, no age limits (children and adults)	Evaluation of public health programs aiming to prevent, control or reduce obesity or obesity‐related risk factors; duration of >3 months	Required control group but not defined	Any anthropometric measure	Required control group (no other limits)	Comprehensive interventions with at least PA, dietary intervention and health education may be effective in reducing obesity in Chinese children.
Godin, 2015^43^	7 (5)	2003–2014	Narrative synthesis	Ages not specified, studies targeting First Nations, Inuit or Metis youth in Canada	School based intervention (elementary or secondary) or ≥ one component implemented in school setting	Not defined	Any anthropometric outcome *(dietary intake, PA)*	Not specified	The majority of interventions did not result in significant improvements in obesity, PA or healthy eating outcomes among First Nations Inuit or Metis youth.
Hillier‐ Brown, 2014^44^	24 (11)	Inception to 2012	Narrative synthesis	0–18 year olds	Prevention and treatment interventions that might reduce socioeconomic inequalities in the prevalence of obesity‐related outcomes; ≥12 weeks duration	Non treatment or standard treatment control group	Any anthropometric outcome	RCT; non‐RCT	Some individual and community based interventions may be effective in reducing socio‐economic inequalities in obesity‐related outcomes among children. However, the evidence was limited.
Hodder, 2022^45^	195 (58)	1990–2021	Meta‐analysis and narrative synthesis	6–18 year olds	Interventions with a rationale to prevent child obesity; post‐intervention outcomes ≥12 weeks from baseline	Nonintervention; usual care; alternative intervention	Any anthropometric outcome *(adverse effects, absolute cost, cost‐effectiveness)*	RCT	Small beneficial effect on BMI for school‐based obesity prevention interventions.
Hung, 2015^46^	27 (19)	Not reported	Meta‐analysis and narrative synthesis	6–18 year olds	School‐based; obesity prevention programs	Required control group (not defined)	BMI or skinfold thickness	RCT; other intervention with control group	School‐based interventions have not been effective for improving BMI or skinfold thickness.
Katz, 2008^47^	Narration: 19 (15) Meta‐analysis: 8 (8)	1966–2004	Meta‐analysis and narrative synthesis	3–18 year olds	School setting; ≥6 months duration; aimed to prevent weight gain or manage weight; interventions related to nutrition, PA, reduction in TV viewing	Include a control measurement (pre/post‐measures or control group)	Any anthropometric outcome	RCT; non‐RCT; pre‐post design	Combination nutrition and PA interventions are effective at achieving weight reduction in school settings.
Kesten, 2011^48^	30 (17)	1990–2010	Narrative synthesis	7–11 year olds; included only girls, or girls only stratification	Prevention intervention; ≥12 weeks; combination of nutrition, PA, knowledge, attitudes or health‐related behaviors	Not defined	Any anthropometric outcomes *(dietary behaviors, PA)*	RCT; controlled pre‐post design; noncontrolled studies;	Majority of interventions did not produce medium to large effect sizes over the long term in a broad range of behavioral and physical measures
Knowlden, 2013^49^	10 (10)	2001 to 2012	Narrative synthesis	Ages not specified; studies targeting African American or Hispanic children	Prevention of overweight/obesity; conducted in U. S schools	Not defined	Any anthropometric outcomes (*Blood pressure, academic scores, PA, self‐efficacy, dietary behaviors)*	Experimental; quasi‐experimental	Efficacy of school‐based interventions targeting minorities can be enhanced by including behavioral theories, systematic process evaluation, long‐term follow‐up, family and home environments.
Korn, 2018^50^	13 (13)	1990–2018	Narrative synthesis	0–12 year olds, studies conducted in high‐income countries	Multisetting, community based obesity prevention that engaged a community coalition in the research process	Not defined	Any anthropometric outcome *(behavioral or environmental/policy)*	Not cross‐sectional or qualitative	Leadership roles of coalitions are important in building relationships, structures, and capacity to implement strategies promoting children's healthy behaviors and weight status.
Kornet‐van der Aa, 2017^51^	14 (9)	2000–2016	Narrative synthesis	12–18 year olds; from low SEP backgrounds	Obesity intervention (prevention or treatment)	Not defined (not required if pre‐post trial)	BMI, BMI *z* score *(PA, dietary intake, sedentary behavior, screen time)*	RCT; non RCT; pre‐post design without control group	No conclusive evidence for which specific intervention strategies were effective in preventing obesity among disadvantaged adolescents
Kropski, 2008^52^	14(11)	1990–2005	Narrative synthesis	‘school children’ (ages not specified); normal‐weight and overweight children	Conducted within schools; curricular and or/environmental (not extra‐curricular); evaluation ≥6 months post baseline	Not defined	Any anthropometric outcome	Experimental; quasi‐experimental	The small number of published studies and methodological concerns limited conclusions being drawn regarding the efficacy of school‐based obesity prevention studies.
Langford, 2014^53^	67 (15)		Meta‐analysis and narrative synthesis	4–18 year olds; attending schools/colleges	Interventions based on the Health Promoting Schools framework—with active engagement in each of (1) school curriculum; (2) ethos or environment of school; (3) families or communities	Schools implementing no intervention/alternative intervention including only one or two of the Health Promoting Schools criteria	Any anthropometric outcome *(dietary intake, PA, tobacco, alcohol, drug, sexual health, mental health, violence, bullying)*	RCTs with clusters at school, district or other geographical area level (not classroom level)	Holistic school‐based interventions, like the Health Promoting Schools framework, can be effective at improving a number of health outcomes in students, especially BMI.
Laws, 2014^54^	32 (14)	1993–2013	Narrative synthesis	0–5 year olds; participants from low SEP or Indigenous backgrounds	Interventions aimed at prevention of unhealthy weight gain and/or obesity related behaviors	Control group, nonequivalent control group or baseline level	Any anthropometric outcomes *(PA, sedentary behaviors, parental feeding practices)*	RCTs; quasi‐experimental; before and after studies	Further research needed on obesity prevention interventions for Indigenous children. Obesity prevention interventions among young children from socioeconomically disadvantaged families appear modest but promising
Liu, 2019^55^	50 (11)	1990–2019	Meta‐analysis and narrative synthesis	5–18 year olds	School based; Prevention of overweight/obesity; ≥3 months duration	Comparison group: active controls, usual practice controls, wait‐list controls	BMI, BMI *z* score	Individual or cluster‐RCT	School‐based interventions generally effective for reducing weight gain in children, but more high‐quality studies needed.
Nally, 2021^56^	Narration: 48 (33) Meta‐analysis: 38(31)	2009–2020	Meta‐analysis and narrative synthesis	5–12 years olds, attending primary school full‐time.	School based interventions; targeting a change in BMI and/or obesity related behaviors (PA, sedentary behavior, diet)	No intervention, alternative treatment condition, usual care	BMI *(dietary intake, PA, sedentary behaviors)*	RCT; cluster‐RCT with comparison or control arm	Meta‐analysis results indicate that school‐based interventions had a small significant effect in BMI (kg/m2) and BMI *z* score compared with controls.
Narzisi, 2021^57^	30 (6)	Inception to 2018	Narrative synthesis	0–5 year olds	Lifestyle intervention targeting ≥2 behaviors (diet, PA, sedentary behavior, sleep, screen time)	Not defined	Any anthropometric outcome	RCT; cluster RCT; single‐blind trial; double‐blind trial; pre‐post test	Multilevel studies were more likely to be effective; however, there is a need for more accessible, multicomponent studies to include low‐income families.
Singhal, 2020^58^	Narration: 21 (14) Meta‐analysis: 9 (6)	Inception to 2019	Meta‐analysis and narrative synthesis	4–12 year olds, with weight status reflective of general child population	Interventions targeting diet and/or PA; primary school‐based; (or equivalent) conducted in middle‐income countries	Control group, no intervention/usual school‐based activities	Any anthropometric outcomes *(dietary intake, PA, quality of life)*	RCT; cluster RCT	Some evidence to support school‐based interventions in preventing childhood obesity in middle‐income countries.
Sobol‐Goldberg, 2013^59^	32 (16)	2006–2012	Meta‐analysis and narrative synthesis	5–18 year olds	School‐based interventions; focused on obesity prevention	Control group not receiving intervention	BMI	RCT	More recent studies showed evidence that school‐based prevention interventions are mildly effective in reducing BMI in children.
Specchia, 2018^60^	Narration: 14 (14) Meta‐analysis: 11(11)	Inception to 2015	Meta‐analysis and narrative synthesis	Under 18 years	Highly integrated obesity prevention program (multicomponent, multilevel, multiple settings)	Not defined	Any anthropometric outcome *(dietary intake, PA, sedentary behavior)*	Not defined	Coordinated cross‐sectoral, multicomponent and multistakeholder initiatives to oppose obesity remain a challenge.
Uijtdewilligen, 2016^61^	17 (10)	Inception to 2015	Narrative synthesis	0–18 year olds	Interventions aimed at reducing and/or preventing obesity	Not defined	Anthropometric outcomes *(energy‐balance related behaviors, psychosocial constructs)*	Not defined	A comprehensive multisectoral approach in preschool settings may increase the effectiveness and sustainability of childhood obesity prevention programs.
Verjans‐Janssen, 2018^62^	25 (18)	Not reported	Narrative synthesis	4–12 year olds, participants attending primary school	School‐based intervention; with ≥1 of changes to school (1) physical environment; (2) social environment; (3) policies; (4) economic support AND directly involved parents	Not defined	BMI, BMI *z* score *(dietary intake, PA, sedentary behavior)*	Not defined	School‐based interventions with direct parental involvement have the potential to improve children's weight status, PA and sedentary behavior.
Verstraeten, 2012^63^	25 (6)	1990–2011	Narrative synthesis, effect sizes calculated for comparison (no meta‐analysis)	6–18 year olds	School‐based interventions targeting dietary behavior and/or PA for the primary prevention of obesity	Required control group, but not defined	Any Anthropometric outcome, *(dietary intake, PA)*	Controlled trial design (with or without randomization)	In low‐ and middle‐income countries, school‐based interventions can potentially improve dietary and PA behavior and prevent unhealthy body weights.
Wang, 2015^64^	139 (88)	Inception to 2013	Meta‐analysis and narrative synthesis	2–18 year olds; conducted in high‐income countries	Obesity prevention; duration ≥1 year follow‐up (≥6 months if school based)	Not defined	Any anthropometric outcome	RCT; quasi‐experimental; natural experiments	Moderately strong evidence supports the effectiveness of school‐based interventions for preventing childhood obesity.
Wolfenden, 2014^13^	Narration: 8 (8) Meta‐analysis 6(6)	1990–2011	Meta‐analysis and narrative synthesis	Community samples of children and/or adults or specific population groups within a community	Population‐based whole of community intervention that: (1) primarily sought to prevent population weight gain, (2) targeted ≥1 determinant of population weight gain, (3) included community consultation or engagement to inform intervention development or delivery	Comparison group receiving no intervention or “treatment as usual,” attention controls or waitlist controls	Any anthropometric outcomes	RCT; cluster RCT; quasi‐experimental designs with control group	Population‐based, whole of community interventions can achieve modest reductions in population weight gain among children.
Zhou, 2014^65^	15 (10)	2000–2012	Narrative synthesis	Childcare facilities or preschool	Interventions aimed at childhood obesity prevention	Not defined	Any anthropometric outcomes *(dietary intake, PA)*	RCT; non‐RCT	Mixed results, with high heterogeneity in studies. Long‐term follow‐up of multistrategy interventions that make changes in the nutrition and PA environment, report cost data, and consider sustainability is needed.

Abbreviations: BMI, body mass index; COI, conflict of interest; HPS, Health Promoting Schools; PA, physical activity; RCT, randomized controlled trial.

^a^ Where multiple publications on the same intervention were included, only the number of interventions are reported.

The included systematic reviews reported on a total of 686 studies that were within the scope of this umbrella review. This included 293 unique primary studies, of which 173 (59%) were included in only one eligible systematic review, and 120 included in two or more systematic reviews.

Table 3 summarizes the practice‐relevant data within the three domains, including the number and proportion of systematic reviews reporting each category (see Table S1 for detailed version for each included systematic review). Reviews that stated an intention to extract information but were not able to due to lack of primary data are noted in Table S3.

**TABLE 3:** Summary of number and percentage of studies reporting categories of data within each domain.

		Not reported *N* (%)	Partially reported *N* (%)	Reported *N* (%)	Reported and analyzed *N* (%)
Intervention characteristics	Intervention behaviors	0 (0%)	0 (0%)	16 (40%)	24 (60%)
	Specific strategies	13 (33%)	8 (20%)	17 (43%)	2 (5%)
	Duration	3 (7.5%)	1 (2.5%)	20 (50%)	16 (40%)
	Intensity	16 (40%)	12 (30%)	11 (27.5%)	1 (2.5%)
	Settings	1 (2.5%)	0 (0%)	24 (60%)	15 (37.5%)
	Organizations or contributors involved	19 (47.5%)	5 (12.5%)	10 (25%)	6 (15%)
Outcome reporting	Effectiveness summarized	0 (0%)		21 (52.5%) narratively 19 (47.5%) quantitatively and narratively	
	Harms	32 (80%)		8 (20%)	
	Sustainability	19 (47.5%)		21 (52.5%)	
	Equity considerations	22 (55%)	7 (17.5%)	6 (15%)	5 (12.5%)
Translation, validity, replicability	Community characteristics	4 (10%)	23 (57.5%)	13 (32.5%)	1 (2.5%)
	Participant characteristics	4 (10%)	20 (50%)	16 (40%)	
	Resource use or costs	33 (82.5%)		7 (17.5%)	

*Intervention characteristics*: All systematic reviews reported which behaviors were targeted by the intervention (e.g., diet, physical activity, sedentary behavior). This was commonly used for stratification purposes, with 60% of reviews providing analysis according to the behavioral targets of the included interventions.^12^, ^29^, ^30^, ^31^, ^32^, ^33^, ^34^, ^36^, ^38^, ^39^, ^40^, ^45^, ^46^, ^47^, ^51^, ^52^, ^53^, ^55^, ^56^, ^58^, ^59^, ^63^, ^64^, ^65^ Specific strategies or elements involved in the intervention, such as policy change, curriculum change, or alterations to the environment, were reported in detail in 17 (43%) systematic reviews^12^, ^13^, ^29^, ^32^, ^34^, ^37^, ^39^, ^41^, ^43^, ^44^, ^50^, ^51^, ^53^, ^56^, ^58^, ^61^, ^63^ and partially reported in eight (20%) systematic reviews.^36^, ^38^, ^40^, ^48^, ^49^, ^52^, ^55^, ^65^ Only two (5%) systematic reviews provided analysis according to specific intervention strategies.^31^, ^33^

Almost all systematic reviews (*n* = 36, 90%) reported the duration of the included interventions,^12^, ^13^, ^28^, ^29^, ^30^, ^31^, ^32^, ^33^, ^34^, ^35^, ^36^, ^37^, ^38^, ^40^, ^41^, ^42^, ^44^, ^45^, ^46^, ^47^, ^48^, ^49^, ^50^, ^51^, ^52^, ^53^, ^55^, ^56^, ^57^, ^58^, ^59^, ^60^, ^61^, ^62^, ^63^, ^65^ with 16 (40%) of these also examining variation in results according to duration (e.g., interventions of less than 12 months compared with those lasting 12 months or more).^29^, ^31^, ^32^, ^33^, ^34^, ^36^, ^37^, ^44^, ^45^, ^46^, ^48^, ^56^, ^58^, ^59^, ^62^, ^63^ Reporting of intervention intensity varied, and was only comprehensively described by 12 reviews (30%).^30^, ^31^, ^32^, ^33^, ^34^, ^37^, ^41^, ^55^, ^58^, ^61^, ^63^, ^65^ This can be a difficult metric to quantify for multicomponent or complex interventions. Examples of some reporting included a 30‐min physical activity session three times a week, three face‐to‐face sessions per week. There was little information on intensity of community input into interventions.

All but one systematic review^60^ included information on the setting of the interventions (e.g., school and community), with 15 (37.5%) using this variable to stratify study results.^12^, ^29^, ^30^, ^34^, ^39^, ^44^, ^45^, ^46^, ^47^, ^48^, ^54^, ^58^, ^59^, ^62^, ^64^ Sixteen (40%) systematic reviews reported on the organizations or contributors involved in the interventions,^13^, ^31^, ^32^, ^33^, ^34^, ^36^, ^39^, ^41^, ^46^, ^50^, ^53^, ^54^, ^58^, ^61^, ^63^, ^65^ with six (15%) of these conducting analyses according to this metric.^31^, ^33^, ^39^, ^46^, ^50^, ^58^ Systematic reviews variously described organizations or contributors involved in the conception, planning, or implementation stage of the interventions. A number of systematic reviews focused on school‐based interventions reported on who had delivered the intervention (often teachers who had been provided with training). Few nonschool‐based systematic reviews reported on the range of organizations or contributors involved in the planning, implementation, or evaluation of the intervention.

*Outcome reporting*: All systematic reviews included a summary of the intervention effectiveness across their included studies. This was done either solely narratively (*n* = 21), or both narratively and quantitatively through meta‐analysis (*n* = 19). Many of the meta‐analyses also included subgroup analysis to investigate the impact of different aspects such as duration, behaviors targeted, and settings on intervention effectiveness.

Harms or adverse effects were only reported by eight (20%) included systematic reviews^32^, ^34^, ^42^, ^45^, ^53^, ^54^, ^58^, ^63^ but were included in some of the discussion sections as an area for improvement.^32^, ^35^, ^42^, ^64^ Just over half (*n* = 21, 52.5%) reported on sustainability of the interventions (i.e., reported outcomes at an additional time point after intervention completion),^12^, ^28^, ^29^, ^31^, ^33^, ^34^, ^38^, ^40^, ^41^, ^45^, ^49^, ^50^, ^51^, ^53^, ^56^, ^57^, ^58^, ^61^, ^62^, ^63^, ^65^ and a lack of sustainability information was also commonly included in the discussion sections of the reviews.

Twenty‐three (57.5%) systematic reviews did not include any information on equity considerations.^12^, ^13^, ^28^, ^29^, ^31^, ^33^, ^35^, ^36^, ^37^, ^39^, ^45^, ^46^, ^47^, ^48^, ^50^, ^52^, ^55^, ^56^, ^59^, ^60^, ^61^, ^62^, ^64^ Of those that did include this information, seven (17.5%) reported equity related information regarding the groups at baseline^29^, ^30^, ^38^, ^57^, ^58^, ^63^, ^65^ (e.g., SEP level and ethnicity) and four (10%) reported differential impacts according to an equity related metric such as SEP levels or ethnicity.^34^, ^40^, ^44^, ^53^ Five systematic reviews explicitly focused on children considered low SES^44^, ^51^ or a particular ethnic group.^32^, ^43^, ^49^ None of these systematic reviews reported using the Preferred Reporting Items for Systematic Reviews and Meta‐Analysis—Equity (PRISMA‐E) to guide their methods.^66^ Similar to the lack of information on costs, the absence of equity considerations in the primary studies was mentioned in the discussion section of four SRs^31^, ^37^, ^55^, ^64^ and had been a predefined aim in one systematic review,^36^ that the authors could not address due to a lack of reporting in primary studies.

*Translation, replicability and validity*: There was variation in how the characteristics of the community were reported. Thirty‐six (90%) systematic reviews included the country the intervention was based,^12^, ^13^, ^28^, ^29^, ^30^, ^31^, ^32^, ^33^, ^34^, ^35^, ^37^, ^38^, ^39^, ^40^, ^41^, ^42^, ^43^, ^44^, ^45^, ^46^, ^47^, ^48^, ^49^, ^50^, ^51^, ^53^, ^54^, ^55^, ^56^, ^57^, ^58^, ^60^, ^61^, ^62^, ^63^, ^65^ while 12 (30%) of these included additional information such as ‘low SEP area’ or ‘rural area’.^29^, ^31^, ^32^, ^34^, ^35^, ^41^, ^43^, ^44^, ^49^, ^54^, ^58^, ^65^ Four (10%) included systematic reviews did not report any details on the participants of included studies,^33^, ^35^, ^59^, ^64^ while 19 (47.5%) reported age and/or sex,^12^, ^13^, ^28^, ^29^, ^36^, ^37^, ^42^, ^46^, ^47^, ^48^, ^50^, ^52^, ^53^, ^55^, ^56^, ^60^, ^61^, ^62^, ^63^ and 16 (40%) included at least one additional factor such as individual level SEP or ethnicity.^30^, ^31^, ^32^, ^34^, ^38^, ^39^, ^40^, ^41^, ^43^, ^44^, ^49^, ^51^, ^54^, ^57^, ^58^, ^65^ The resources required, costs, and/or cost effectiveness of the interventions were included in seven (17.5%) systematic reviews,^32^, ^34^, ^38^, ^42^, ^45^, ^53^, ^54^ although further detail was generally lacking regarding this. Reporting information on costs was a predefined aim in one systematic review^63^; however, the authors reported that this information was not available in any of the primary studies. The discussion section of nine systematic reviews^36^, ^40^, ^41^, ^44^, ^52^, ^58^, ^63^, ^64^, ^65^ included comment on the lack of information on costs or cost effectiveness of interventions in primary papers.

### 3.1 Quality assessment tool—AMSTAR‐2

The outcomes of the AMSTAR‐2 quality assessment tool are shown in Table 4, with categorization based on the AMSTAR‐2 guidelines.^27^ There was high confidence in the results of two systematic reviews.^34^, ^53^ There was low confidence in the results of 14 systematic reviews,^12^, ^31^, ^35^, ^44^, ^45^, ^51^, ^52^, ^54^, ^55^, ^56^, ^58^, ^59^, ^62^, ^63^ while 24 were assessed as critically low.^13^, ^28^, ^29^, ^30^, ^32^, ^33^, ^36^, ^37^, ^38^, ^39^, ^40^, ^41^, ^42^, ^43^, ^46^, ^47^, ^48^, ^49^, ^50^, ^57^, ^60^, ^61^, ^64^, ^65^ All systematic reviews outlined their inclusion criteria according to participants, intervention, comparison and outcomes (PICO) and 87.5% (*n* = 35) assessed the primary studies using a quality assessment tool.^12^, ^13^, ^29^, ^30^, ^32^, ^33^, ^34^, ^35^, ^36^, ^37^, ^38^, ^39^, ^40^, ^41^, ^42^, ^43^, ^44^, ^45^, ^47^, ^48^, ^51^, ^52^, ^53^, ^54^, ^55^, ^56^, ^57^, ^58^, ^59^, ^60^, ^61^, ^62^, ^63^, ^64^, ^65^ These tools included the Downs and Black Quality Checklist for Healthcare Intervention studies,^67^ the risk of bias tool outlined in the Cochrane Handbook for Systematic Review of Intervention,^68^ and the Effective Public Health Practice Project Quality Assessment Tool.^69^ However, while most used a quality assessment tool, often these results were reported in isolation and not integrated into the narration, conclusions or explanation of heterogeneity in the results. Less than half of systematic reviews (*n* = 17, 42.5%) registered a protocol prior to the undertaking of the study^13^, ^30^, ^32^, ^34^, ^35^, ^44^, ^45^, ^46^, ^50^, ^51^, ^52^, ^53^, ^54^, ^56^, ^58^, ^59^, ^65^ and only four systematic reviews included details (reference, and reason for exclusion) on studies that were excluded at the full text stage,^34^, ^36^, ^53^, ^55^ which was the most common critical flaw among the included systematic reviews.

**TABLE 4:** Results of AMSTAR‐2 critical appraisal tool.^27^

Author, year	1. PICO	2. Prior methods^a^	3. Study design	4. Literature search^a^	5. Duplicate study selection	6. Duplicate data extraction	7. Excluded studies justified^a^	8. Included studies described?	9. Risk of bias (RoB)^a^	10. Funding of primary studies	11. Meta‐analysis ‐ methods^a^	12. Meta‐analysis‐ impact of RoB	13. Accounting for RoB in results^a^	14. Discussion of heterogeneity results	15. Publication bias^a^	16. COI/funding for review	Overall confidence
Angawi, 2021^28^																	CL
Bleich, 2013^12^																	L
Bleich, 2018^29^																	CL
Bramante, 2019^30^																	CL
Brown, 2009^31^																	L
Brown, 2015^32^																	CL
Brown, 2016^33^																	CL
Brown, 2019^34^																	H
Buchanan, 2023^35^																	L
Campbell, 2001^36^																	CL
Cerrato‐Carretero, 2021^37^																	CL
Chavez, 2020^38^																	CL
Connelly, 2007^39^																	CL
Dabravolskaj, 2020^40^																	CL
De Bourdeaudhuij, 2009^41^																	CL
Goa, 2008^42^																	CL
Godin, 2015^43^																	CL
Hillier‐Brown, 2014^44^																	L
Hodder, 2022^45^																	L
Hung, 2015^46^																	CL
Katz, 2008^47^																	CL
Kesten, 2011^48^																	CL
Knowlden, 2013^49^																	CL
Korn, 2018^50^																	CL
Kornet‐van der Aa, 2017^51^																	L
Kropski, 2008^52^																	L
Langford, 2014^53^																	H
Laws, 2014^54^																	L
Liu, 2019^55^																	L
Nally, 2021^56^																	L
Narzisi, 2021^57^																	CL
Singhal, 2020^58^																	L
Sobol‐Goldberg, 2013^59^																	L
Specchia, 2018^60^																	CL
Uijtdewilligen, 2016^61^																	CL
Verjans‐Janssen, 2018^62^																	L
Verstraeten, 2012^63^																	L
Wang, 2015^64^																	CL
Wolfenden, 2014^13^																	CL
Zhou, 2014^65^																	CL

Abbreviations: COI, conflict of interest; Green, Yes; Orange, Partial Yes; Red, No; PICO, Participants, Intervention, Comparison, Outcome; RoB, Risk of Bias (see Table S4 for explanation of each criterion).

^a^ Critical domain. Result is a rating of overall confidence in the results of the review^27^—graded as follows: CL: critically low (more than one critical flaw with or without noncritical weaknesses); L: low (one critical flaw with or without noncritical weaknesses); M: moderate (more than one noncritical weakness, but no critical flaws); H: high (no critical flaws and one or none noncritical weakness).

## 4 DISCUSSION

In this umbrella review, we sought to address the question of whether, and to what extent, published systematic reviews include important detail to inform decisions regarding investment in implementation and further development of community‐based childhood obesity prevention interventions. We found that systematic reviews commonly report on some essential elements related to the characteristics and evaluation of community‐based childhood obesity prevention interventions, including outcome measures, strategies employed, the settings within which these were implemented and the duration of the interventions. Other characteristics of interventions that could be useful for decision‐makers, such as the role of different organizations or contributors, the intensity of interventions, heterogeneity in effectiveness across different population groups, sustainability of interventions, potential harms, and costs of the intervention were less commonly reported. When these factors were reported they were often reported descriptively, without any analysis or critique. Concerningly, appraisal using the AMSTAR‐2 tool found low or critically low confidence in the results reported in most of the systematic reviews included in this study.

Recent studies have demonstrated the importance of the local context in all stages of intervention design, delivery, and evaluation,^10^, ^70^ yet our findings suggest that systematic reviews provide relatively limited data to assist decision‐makers in achieving these goals, and identifying strong evidence for interventions that are likely to be effective within their context. Data on local context have been shown to be a critical aspect of the design and implementation of community based interventions; however, our findings suggest that decision‐makers would not be able to rely on the synthesized evidence as it does not comprehensively cover many important factors.

Of particular note, the reviews included in our umbrella review rarely reported the impacts of CBIs according to important equity subgroups. This reflects a lack of equity analyses and reporting in primary studies and a missed opportunity for tackling inequities in health. While there is widespread recognition that understanding differential impacts of CBIs is crucial for policy making, it is difficult to do so, in a statistically appropriate manner (requiring much larger sample sizes for powered analysis), without conducting very large trials, or combining the individual participant data from a number of comparable trials. The many different types of important subgroups add further complexity. To ensure equity considerations are front and center of CBI analysis and reporting, researchers and practitioners should work with priority populations in communities to understand how equity can be incorporated into evaluation frameworks early in their design. A range of evidence should also be considered, including qualitative data to reflect the lived experiences of identified priority groups. The PROGRESS‐PLUS framework may be used to guide which priority groups to focus on.^71^

Only seven (17.5%) of the systematic reviews included in our umbrella review reported on the resource use, costs or cost‐effectiveness of CBIs, despite recognition that this is important information to enhance review usefulness and applicability to decision‐making.^72^ Clearly there is more scope for the inclusion of economic evidence into systematic reviews of CBIs for obesity prevention with a focus on practice‐relevant process or implementation factors. The limited economic evidence incorporated into the systematic reviews in our umbrella review is also however, reflective of the limited primary economic evidence base for the resource use, costs and cost‐effectiveness of CBIs for obesity prevention. A recent systematic review identified only 17 studies reporting economic evidence for obesity prevention CBIs, based on 13 different interventions worldwide.^11^ Only five CBIs had been subjected to a full economic evaluation (assessing both the costs and benefits of a CBI compared with a comparator), five CBIs had published protocols for full economic evaluation, two CBIs reported cost analyses and one CBI reported a costing protocol.^11^ While the challenges of conducting economic studies alongside CBIs are well recognized,^73^ and include the complexity of ascertaining costs and effects of multisector, multistrategy interventions, there is a clear need to build the investment case for CBIs for obesity prevention.

The lack of information on elements such as equity and sustainability were commonly included in systematic review discussion sections, pointing to a lack of this information in primary studies. Further, one systematic review that did include a majority of the practice‐relevant information^50^ had contacted the authors of the included primary papers to gather this level of detail. Constraints on publication length by journals may also impact on the amount of information provided. However, those systematic reviews that did provide a significant amount of relevant information utilized supplementary tables and figures to do so, which are generally available with online publications.

Our umbrella review has several strengths. Our search was comprehensive in nature, registered via PROSPERO and followed established umbrella review guidance (PRIO‐harms).^23^ The databases used for our search represent the key data sources in the field and the search terms would be expected to return all community‐based interventions in the published literature. The inclusion criteria and search terms were broader than “community based” and also included studies reported in community settings. The use of a quality assessment tool^27^ provided the means to assess the quality of the conclusions reached by the reviews included in this study.

Our review is limited by a lack of a gold standard for conducting and reporting umbrella reviews. We also did not search gray literature and it is possible that some studies were missed that would have met the inclusion criteria. There may also be other key sources of evidence synthesis (e.g., national health guidelines) that are relevant to decision‐makers and practitioners, but were not included due to our focus on systematic reviews. Our inclusion criteria specified a weight outcome; however, some relevant reviews of CBIs focused on obesity prevention may have specified different outcomes, or have had different indicators of success and were therefore excluded. A further limitation that commonly impacts umbrella reviews is the potential for overlap in the primary studies included in each systematic review.^74^ Therefore, if information is lacking in these publications it is carried across all the systematic reviews. However, it is important for systematic reviews to highlight their a priori aims of extracting particular information, even if it was not available in the primary papers, as was the case in two included systematic reviews.^36^, ^63^ A further limitation was the use of a second independent reviewer for only 10% of the sample when completing the quality assessment AMSTAR‐2 tool.

Our findings suggest much work has been done in CBIs yet large gaps remain in our knowledge about how they work, who makes them work and therefore how we might optimize future interventions, and learn from those that were not effective. The limited reporting of which organizations or contributors are involved in the stages of design, implementation and evaluation provides a salient example. Taking an evidence‐based approach to planning CBIs, and despite there being 40 reviews of these studies, planners would not have clear guidance of who should be involved and at which stage. This is despite repeated calls for strong engagement across multiple organizations or contributors at all stages of CBIs.^75^

Future research is needed to close the gaps in knowledge identified in our umbrella review. Notably the roles and interactions between key actors in CBIs is an understudied area. Several attempts have been made to track these factors, more recently using social network analysis^76^, ^77^ and system mapping software,^78^ though network analysis is often limited by low response rates. Exploration of automated and large data methods for collecting information on the types and timing of organizations or contributors input provides a new area for CBI research. These developments would provide the basis for understanding how collaborator and organizations' behaviors (in networks) then drives actions observed within CBIs, and contribute to intervention effectiveness. Such analyses are in their infancy^76^ but provide the basis for collecting detailed information in a way that can support a generalizable approach to implementation in CBIs. Equity considerations and cost‐benefits should also be embedded into evaluation frameworks from the outset.

## 5 CONCLUSION

Multilevel interventions for child obesity prevention have demonstrated effectiveness, yet additional documentation of successful intervention processes is needed. While potentially limited by the information provided in primary studies, systematic reviews could provide more relevant and useful evidence synthesis through greater focus on the development and intervention characteristics that contribute to CBI success. This may allow decision‐makers to understand what is needed for planning and adapting a CBI to their own context. Establishing generalizable best‐practice approaches to CBIs would capitalize on existing evidence, and ensure that future interventions are optimized for effectiveness.

## CONFLICT OF INTEREST STATEMENT

None to declare.

## Supporting information


**Table S1:** Search terms.
**Table S2:** Domains and responses.
**Table S3:** Reporting of practice‐relevant information in included systematic reviews.
**Table S4:** Detail of AMSTAR‐2 rating criteria.
