# Supplementary material for: Umbrella review of systematic reviews to inform the development and translation of community‐based childhood obesity prevention interventions
Source: Obes Rev. 2024 Nov 23;26(3):e13864. doi: 10.1111/obr.13864 (PMC11791390; doi:10.1111/obr.13864)
Supplement: Supplementary file 1 — Table S1: Search terms. Table S2: Domains and responses. Table S3: Reporting of practice‐relevant information in included systematic reviews. Table S4: Detail of AMSTAR‐2 rating criteria. [file OBR-26-e13864-s001.docx]

**Supporting Information**

**Title:** Umbrella review of systematic reviews to inform the development and translation of community-based childhood obesity prevention interventions

**Authors:**

Jane Jacobs^1^, Luke Wolfenden^2^, Kristy A Bolton^1,3^, Vicki Brown^1,4^, Marufa Sultana^1,4^, Kathryn Backholer^1^, Steven Allender^1^, Rachel Novotny^5^, Anna Peeters^1^, Melanie Nichols^1^

^1^ Deakin University, Geelong, Australia, Institute for Health Transformation, Global Centre for Preventive Health and Nutrition, School of Health and Social Development, Faculty of Health

^2^ School of Medicine and Public Health, Faculty of Health and Medicine, University of Newcastle, Callaghan, NSW, 2308, Australia

^3^ Deakin University, Geelong, Australia, Institute for Physical Activity and Nutrition, School of Exercise and Nutrition Sciences, Faculty of Health

^4^ Deakin University, Geelong, Australia, Institute for Health Transformation, Deakin Health Economics, School of Health and Social Development, Faculty of Health

^5^ University of Hawaii at Manoa, College of Tropical Agriculture and Human Resources, Department of Human Nutrition, Food and Animal Sciences

**Corresponding Author:**

Jane Jacobs

Deakin University, 1 Gheringhap st, Geelong, 3220, Victoria, Australia

[jane.jacobs@deakin.edu.au](mailto:jane.jacobs@deakin.edu.au)

**Table S1**: Search terms used for Medline Complete (via EBSCOhost)

|  | **Concept** | **Search Terms (Medline)** |
| --- | --- | --- |
| 1 | Systematic review | ‘Systematic review’ OR ‘Cochrane review’ OR ‘meta-analy*’ |
| 2 | Prevention | (MH "primary prevention") OR prevent* |
| 3 | Intervention | intervention OR (MH "Program Evaluation") OR evaluat* OR initiative OR program* OR strateg* OR project* |
| 4 | Obesity | (MH ‘obesity’) OR (MH ‘pediatric obesity’) OR (MH ‘overweight’) OR (MH ‘ Waist-Hip Ratio’) OR (MH ‘Body Mass Index’) OR (MH ‘ adiposity’) OR (MH ‘Anthropometry’) OR (MH ‘ Waist Circumference’) OR obes* OR overweight OR ‘body mass index’ OR bmi OR weight OR waist OR adipos* OR anthropometry OR ‘fat mass’ OR ‘skinfold thickness’ OR zBMI OR ‘growth trajectory’ |
| 5 | Children and adolescents | (MH ‘Child’) OR (MH ‘ adolescent’) OR (MH ‘ Child, preschool’) OR (MH ‘pediatrics’) OR child* OR adolesc* OR preschool* OR boy* OR girl* OR young* OR youth OR pediatric OR paediatric OR teen* OR baby OR toddler OR infant |

1 AND 2 AND 3 AND 4 AND 5

| **Table S2:** Detailed description and responses for each category assessed to inform Table 3 | | | |
| --- | --- | --- | --- |
|  | Category | Description | Response categories |
| **Intervention characteristics** | Intervention/ behavioural target reported (i.e PA, diet etc) | Did the systematic review provide information on the behavioural targets of primary studies (e.g PA, diet, sedentary behaviour) | 1. Not reported;  2. Partially reported;  3. Reported/described in a table or narration;  4. Reported and analysed (must include variation by this factor. E.g compare duration, (6 months or less/more than 6 months) number of behaviours targeted (2 behaviours/ more than 2 behaviours)) |
|  | Specific strategies/elements reported | Did the systematic review include adequate information on the specific strategies implemented, beyond behaviours targeted. (e.g policy, curriculum etc) |  |
|  | Duration of intervention reported | Did the systematic review include the length of the intervention (weeks, months, years) |  |
|  | Intensity (frequency of activities, staff time, resources) reported | Did the systematic review include information on how often elements of the intervention were conducted, how much time was required by those implementing the intervention |  |
|  | Number or range of settings involved in interventions reported | Did the systematic review report on which settings the intervention took place in. e.g school, pre-school, community centres, home |  |
|  | Number or range of stakeholders involved in interventions reported | Did the systematic review report on who was involved in the development, implementation, follow-up of the intervention. e.g. community members, teachers, researchers, dieticians |  |
| **Outcome Reporting** | Intervention effectiveness summarised across studies | Did the systematic review include a narrative or quantative summary of effectiveness on an anthropometric outcome of the included whole-of-community or school studies | 1. Not reported (no overall summary included)  2. Reported (narratively; quantitatively and narratively) |
|  | Harms or adverse effects | Did the systematic review report on harms or adverse effects | 1. Not reported (no information provided)  2. Reported (information reported on whether the primary studies include consideration of harms or adverse effects) |
|  | Sustainability of the intervention | Did the systematic review report on whether sustainability of the intervention was included (assessed as including length of intervention and follow-up time). | 1. Not reported (outcomes only reported at end of intervention)  2. Reported (outcomes reported at the end of intervention, and at a time-point after the end of the intervention) |
|  | Equity impacts and considerations | Did the systematic review include information on differential impacts of the intervention on different groups (e.g low vs high SEP, rural vs metro). Or did the SR have a focus on a particular minority or high risk group | 1. Not reported (no information on equity impacts/ considerations)  2. Partially reported (information provided on an equity aspect (described), but no differential analysis  3. Reported (review focused on particular group (e.g low SEP, African-American youth))  4. Reported and analysed (information on differential outcomes according to an equity aspect reported (e.g low vs high SEP)) |
| **Translation, replicability and validity** | Characteristics of the 'community' reported (e.g location, SEP rurality) | Did the systematic review include characteristics of the community from which the sample was taken (e.g Country, city, SES, rurality) | 1.No characteristics reported;  2.Partially reported (Country/city location only reported)  3. Reported (country/city and at least one other characteristic reported (e.g rurality, area-level SEP)  4. Reported and analysed (included variation by a community characteristic e.g. results reported by continent) |
|  | Characteristics of the sample (participants) | Did the systematic review report characteristics of the participants in the primary studies | 1. No participant characteristics reported  2. Partially reported (age and/or sex reported)  3. Reported (age/sex and at least one other characteristic reported (e.g race, individual level SEP)) |
|  | Required resources or intervention costs reported | Did the systematic review include information on the required resources or intervention costs | 1. Not reported  2. Reported (information on resources or cost or cost effectiveness reported) |

Table notes PA: physical activity; SEP: socio-economic position

| **Table S3:** Reporting of practice relevant factors by included systematic reviews | | | | | | | | | | | | | |  |
| --- | --- | --- | --- | --- | --- | --- | --- | --- | --- | --- | --- | --- | --- | --- |
|  | **Intervention characteristics** | | | | | | **Outcome reporting** | | | | **Translation, replicability and validity** | | |  |
| **Author, year** | ***Intervention target*** | ***Specific strategies*** | ***Duration*** | ***Intensity*** | ***Setting*** | ***Organisations or contributors involved*** | ***Effectiveness summary*** | ***Harms*** | ***Sustainability*** | ***Equity considerations*** | ***Community characteristics*** | ***Participant characteristics*** | ***Cost/ cost effectiveness*** |  |
| Angawi, 2021^28^ | Reported | Not reported | Reported | Not reported | Reported | Not reported | Narrative summary | Not reported | Reported | Not reported | Country reported | Age reported | Not reported |  |
| Bleich, 2013^12^ | Reported and analysed | Reported | Reported | Not reported | Reported and analysed | Not reported | Narrative summary | Not reported | Reported | Not reported | Country reported | Age, sex reported | Not reported |  |
| Bleich, 2018^29^ | Reported and analysed | Reported | Reported and analysed | Partially reported | Reported and analysed | Partially reported | Narrative summary | Not reported | Reported | Not reported | Country, city, area level SEP, rurality reported | Age, sex reported | Not reported |  |
|  |  |  |  |  |  |  |  |  |  |  |  | (analysed outcomes according to sex) |  |  |
| Bramante, 2019^30^ | Reported and analysed | Not reported* | Reported | Reported | Reported and analysed | Not reported | Quantative and narrative summary | Not reported | Not reported* | Equity aspects described | Country reported | Age, sex, ethnicity reported | Not reported |  |
| Brown, 2009^31^ | Reported and analysed | Reported and analysed | Reported and analysed | Reported | Reported | Reported and analysed | Narrative summary | Not reported | Reported | Not reported* | Country, rurality, area level SEP reported | Age, sex, ethnicity individual level SEP reported | Not reported |  |
| Brown, 2015^32^ | Reported and analysed | Reported | Reported and analysed | Reported | Reported | Reported | Quantative and narrative summary | Reported | Reported | Review focused on particular group (South Asian populations) | Country, area level SEP | Sex, age, ethnicity, individual level SEP reported | Reported |  |
| Brown, 2016^33^ | Reported and analysed | Reported and analysed | Reported and analysed | Reported | Reported | Reported and analysed | Narrative summary | Not reported* | Reported | Not reported | Country reported | Not reported | Not reported |  |
| Brown, 2019^34^ | Reported and analysed | Reported | Reported and analysed | Reported | Reported and analysed | Reported | Quantative and narrative summary | Reported | Reported | Reported and described strategies to address disadvantage/diversity | Place, Race, Occupation, Gender, Religion, Education, SEP, Social status reported | Age, sex, individaul level SEP, ethnicity reported | Reported |  |
| Buchanan, 2023^35^ | Reported | Not reported | Not reported | Not reported | Reported | Not reported | Quantative and narrative summary | Not reported^ | Not reported* | Not reported^$^ | Country and rurality reported | Not reported^$^ | Not reported |  |
|  |  |  |  |  |  |  |  |  |  |  |  |  |  |  |
| Campbell, 2001^36^ | Reported and analysed | Partially reported | Reported and analysed | Partially reported | Reported | Reported | Narrative summary | Not reported | Not reported* | Not reported^ | Not reported | Age, sex reported | Not reported* |  |
| Cerrato-Carretero, 2021^37^ | Reported | Reported | Reported and analysed | Reported | Reported | Partially reported | Quantative and narrative summary | Not reported | Not reported | Not reported* | Country reported | Age, sex reported | Not reported |  |
| Chavez, 2020^38^ | Reported and analysed | Partially reported | Reported | Partially reported | Reported | Partially reported | Quantative and narrative summary | Not reported | Reported | Information provided on equity aspect (SEP) | Country/ city reported | Age, sex and individual-level SEP reported | Reported |  |
| Connelly, 2007^39^ | Reported and analysed | Reported | Partially reported | Partially reported | Reported and analysed | Analysed only | Narrative summary | Not reported | Not reported | Not reported | Country reported | Age, sex, ethnicity reported | Not reported |  |
| Dabravolskaj, 2020^40^ | Reported and analysed | Partially reported | Reported | Not reported | Reported | Not reported | Quantative and narrative summary | Not reported | Reported | Information provided on an equity aspect (SEP, ethnicity) | Country reported | Age, sex, ethnicity and individual-level SEP | Not reported* |  |
| De Bourdeaudhuij, 2009^41^ | Reported | Reported | Reported | Reported | Reported | Reported | Narratively and in table grouped by intervention approach | Not reported | Reported | Information provided on equity aspect (SEP, ethnicity) | Country, city, rurality reported | Age, sex, ethnicity, individual- level SEP | Not reported* |  |
| Goa, 2008^42^ | Reported | Not reported | Reported | Not reported | Reported | Not reported | Narrative summary | Reported | Not reported* | Not reported | Location within China reported. | Age reported | Reported |  |
| Godin, 2015^43^ | Reported | Reported | Not reported | Partially reported | Reported | Partially reported* | Narrative summary | Not reported | Not reported* | Review focused on particular group (First Nations, Inuit and Metis youth) | Location within Canada and rurality reported | Minority group reported | Not reported |  |
| Hillier-Brown, 2014^44^ | Reported | Reported | Reported and analysed | Not reported | Reported and analysed | Not reported | Narrative summary | Not reported | Not reported* | Review focused on particular group (low SEP) and differential outcomes reported according to an equity aspect (SEP) | Country, area-level SEP reported | Age, sex, individual level SEP reported | Not reported* |  |
| Hodder, 2022^45^ | Reported and analysed | Not reported | Reported and analysed | Not reported | Reported and analysed | Not reported | Quantative and narrative summary | Reported | Reported | Not reported | Country, and analysed by geographical region (continent) | Age, sex, and analysed by age groups | Reported |  |
|  |  |  |  |  |  |  |  |  |  |  |  |  |  |  |
| Hung, 2014^46^ | Reported and analysed | Not reported | Reported and analysed | Not reported | Reported and analysed | Reported and analysed | Quantative and narrative summary | Not reported | Not reported | Not reported | Country, city reported | Age category (>/<10 years) | Not reported |  |
| Katz, 2008^47^ | Reported and analysed | Not reported | Reported | Not reported | Reported and analysed | Not reported | Quantative and narrative summary | Not reported | Partially reported | Not reported | Country, city reported | Age, sex reported | Not reported |  |
| Kesten, 2011^48^ | Reported | Partially reported | Reported and analysed | Partially reported | Reported and analysed | Not reported | Quantative summary (effect sizes), but no overall meta-analysis | Not reported | Not reported* | Not reported | Country reported | Age, sex (focus on girls) reported | Not reported |  |
| Knowlden, 2013^49^ | Reported | Partially reported | Reported | Partially reported | Reported | Not reported* | Narratively | Not reported | Reported | Review focused on particular group (African American or Hispanic children) | Country (all in US) area-level SEP area, rurality reported | Grade, ethnicity reported | Not reported |  |
| Korn, 2018^50^ | Reported | Reported | Reported | Not reported | Reported | Reported and analysed | Narratively | Not reported | Reported | Not reported | Country, city reported | Age/ school year reported | Not reported |  |
| Kornet-van der Aa, 2017^51^ | Reported and analysed | Reported | Reported | Partially reported | Reported | Partially reported | Narratively | Not reported | Reported | Review focused on particular group (low SEP adolescents) | Country, state reported | Age, sex, ethnicity, individual level SEP reported | Not reported |  |
| Kropski, 2008^52^ | Reported and analysed | Partially reported | Reported | Partially reported | Reported | Not reported | Narratively | Not reported | Not reported* | Not reported | Not reported | Age, sex reported | Not reported* |  |
| Langford, 2014^53^ | Reported and analysed | Reported | Reported | Partially Reported | Reported | Reported | Quantative and narrative summary | Reported | Reported | Information on differential outcomes according to equity aspects reported (ethnicity, SEP) | Country reported | Age reported | Reported |  |
| Laws, 2014^54^ | Reported | Not reported | Reported | Not reported | Reported and analysed | Reported | Narratively | Reported | Not reported | Review focused on particular group (children from low SEP or Indigenous backgrounds) | Country, area-level SEP, rurality reported | Age, individual-level SES reported | Reported |  |
| Liu, 2019^55^ | Reported and analysed | Partially reported | Reported | Reported and analysed | Reported | Not reported | Quantative and narrative summary | Not reported | Not reported* | Not reported* | Country reported | Age, sex reported | Not reported |  |
| Nally, 2021^56^ | Reported and analysed | Reported | Reported and analysed | Partially reported | Reported | Not reported | Quantative and narrative summary | Not reported | Reported | Not reported | Country reported | Age, sex reported | Not reported |  |
| Narzisi, 2021^57^ | Reported | Not reported | Reported | Partially reported | Reported | Not reported | Narratively | Not reported | Reported | Information provided on an equity aspect (SEP, ethnicity) | Country reported | Age, ethnicity, individual level SEP reported | Not reported |  |
| Singhal, 2020^58^ | Reported and analysed | Reported | Reported and analysed | Reported | Reported and analysed | Reported and analysed | Quantative and narrative summary | Reported | Reported | Information provided on an equity aspect (SEP, rurality) | Country, city, area-level SEP, rurality reported | Age, sex, individual level SEP reported | Not reported* |  |
| Sobol-Goldberg, 2013^59^ | Reported and analysed | Not reported | Reported and analysed | Not reported | Reported and analysed | Not reported | Quantative and narrative summary | Not reported | Not reported | Not reported | Not reported | None reported | Not reported |  |
| Specchia, 2018^60^ | Reported | Not reported | Reported | Not reported | Not reported | Not reported | Quantative and narrative summary | Not reported | Not reported* | Not reported | Country reported | Age reported | Not reported |  |
| Uijtdewilligen, 2016^61^ | Reported | Reported | Reported | Reported | Reported | Reported | Narratively | Not reported | Reported | Not reported | Country reported | Age, sex reported | Not reported |  |
| Verjans-Janssen, 2018^62^ | Reported | Not reported | Reported and analysed | Not reported | Reported and analysed | Not reported | Narratively | Not reported | Reported | Not reported | Country reported | Age reported | Not reported |  |
| Verstraeten, 2012^63^ | Reported and analysed | Reported | Reported and analysed | Reported | Reported | Reported | Narratively, with summary of effect sizes (no meta analysis) | Reported | Reported | Information provided on an equity aspect (SEP, rurality) | Country, city reported | Age, sex reported | Not reported^* |  |
| Wang, 2015^64^ | Reported and analysed | Not reported | Not reported | Not reported | Reported and analysed | Not reported* | Quantative and narrative summary | Not reported* | Not reported* | Not reported* | Not reported | Not reported | Not reported* |  |
| Wolfenden, 2014^13^ | Reported | Reported | Reported | Not reported | Reported | Reported | Quantative and narrative summary | Not reported | Not reported | Not reported | Country, city reported | Age reported | Not reported |  |
| Zhou, 2014^65^ | Reported and analysed | Partially reported | Reported | Reported | Reported | Reported | Narratively | Not reported | Reported | Information provided on an equity aspect (SEP, ethnicity) | Country, city, area-level SEP reported | Age, ethnicity reported | Not reported* |  |

^ was a pre-specified aim, but not possible with included studies

*included as a discussion point

^$^summary provided, but not in relation to individual studies

**Table S4:** Detail of AMSTAR-2 critical appraisal tool rating criteria

| 1. | Did the research questions and inclusion criteria for the review include the components of PICO? |
| --- | --- |
| 2. | Did the report of the review contain an explicit statement that the review methods were established prior to the conduct of the review and did the report justify any significant deviations from the protocol?* |
| 3. | Did the review authors explain their selection of the study designs for inclusion in the review? |
| 4. | Did the review authors use a comprehensive literature search strategy?* |
| 5. | Did the review authors perform study selection in duplicate? |
| 6. | Did the review authors perform data extraction in duplicate? |
| 7. | Did the review authors provide a list of excluded studies and justify the exclusions?* |
| 8. | Did the review authors describe the included studies in adequate detail? |
| 9. | Did the review authors use a satisfactory technique for assessing the risk of bias (RoB) in individual studies that were included in the review?* |
| 10. | Did the review authors report on the sources of funding for the studies included in the review? |
| 11. | If meta-analysis was performed did the review authors use appropriate methods for statistical combination of results?* |
| 12. | If meta-analysis was performed, did the review authors assess the potential impact of RoB in individual studies on the results of the meta-analysis or other evidence synthesis? |
| 13. | Did the review authors account for RoB in individual studies when interpreting/discussing the results of the review?* |
| 14. | Did the review authors provide a satisfactory explanation for, and discussion of, any heterogeneity observed in the results of the review? |
| 15. | If they performed quantitative synthesis did the review authors carry out an adequate investigation of publication bias (small study bias) and discuss its likely impact on the results of the review?* |
| 16. | Did the review authors report any potential sources of conflict of interest, including any funding they received for conducting the review? |

*critical domain
